# Methodology to Investigate Effect of Prosthetic Interface Design on Residual Limb Soft Tissue Deformation

**DOI:** 10.33137/cpoj.v6i1.42196

**Published:** 2024-01-17

**Authors:** T Arnstein, A Buis

**Affiliations:** Department of Biomedical Engineering, Faculty of Engineering, University of Strathclyde, Glasgow, Scotland.

**Keywords:** Prosthetic, Interface, Socket, MRI, Tissue Strain, Deformation, Lower Limb Amputation, Residual Limb, Rehabilitation, Prosthesis, Tissue Deformation

## Abstract

**BACKGROUND:**

Residual limb discomfort and injury is a common experience for people living with lower limb amputation. Frequently, inadequate load distribution between the prosthetic device and the residual limb is the root cause of this issue. To advance our understanding of prosthetic interface fit, tools are needed to evaluate the mechanical interaction at the prosthetic interface, allowing interface designs to be evaluated and optimised.

**OBJECTIVE:**

Present a methodology report designed to facilitate comprehension of the mechanical interaction between the prosthetic interface and the residual limb. As a pilot study, this methodology is used to compare a hands-on and hands-off interface for a single transtibial prosthesis user using secondary Magnetic Resonance Imaging (MRI) data.

**METHODOLOGY:**

MRI data of the residual limb while wearing a prosthetic interface is segmented into a hard tissue and a skin surface model. These models are exported as stereolithography (STL) files. Two methods are used to analyse the interface designs. Firstly, CloudCompare software is used to compute the nearest vertex on the skin surface for every vertex on the compiled internal bony surface for both interface types. Secondly, CloudCompare software is used to compare registered skin surfaces of the residual limb while wearing the hands-on and hands-off interfaces.

**FINDINGS:**

The maximum and minimum nearest distances between the internal bony surface and skin surface were similar between interface types. However, the distribution of nearest distances was different. When comparing the skin surface while wearing both interfaces, where the fit is more compressive can be visualized. For the dataset used in this study, the classic features of a hands-on Patella Tendon Bearing interface and hands-off pressure cast interface could be identified.

**CONCLUSION:**

The methodology presented in this report may give researchers a further tool to better understand how interface designs affect the soft tissues of the residual limb.

## INTRODUCTION

For people living with amputation, use of a prosthetic device facilitates functional restoration of the missing limb. Ensuring safe load transfer between the prosthetic device and residual limb is critically important in successful rehabilitation. Improper loading at this interface can cause discomfort and injury to the user's residual limb. This is a common experience for people living with lower limb amputation^1–3^ that can result in reduced prosthesis use or device rejection, limiting mobility and hence the ability of users to carry out activities of daily living.^4,5^ Furthermore, to remediate improper fit, additional devices are required to be manufactured placing increased strain on prosthetic care facilities and an additional burden on the user.^1^ As a result, improved interface design is consistently highlighted as a key requirement by prosthesis users worldwide.^6^

The prosthetic interface describes the socket and liner. The geometry and mechanical characteristics of these components play a significant role in dictating how load is transferred between the prosthetic device and residual limb. Typically, a prosthetic socket is a quasi-rigid shell manufactured from a thermosetting or fibre-reinforced polymer. Sitting in between the socket and residual limb, the prosthetic liner is an elastomeric or foam sleeve that allows some level of adaptability to reduce interface pressure concentrations. To accommodate the unique geometry, features, and behaviour of each residual limb, prosthetic sockets must be custom made devices. Two distinct design paradigms exist for prosthetic socket manufacture: hands-on and hands-off casting.^7,8^ Hands-on casting refers to a clinician manually taking a cast of the residual limb and then rectifying this model to transfer load via purportedly load tolerant regions. Within the hands-on category, Computer Aided Design and Manufacture (CAD/CAM) is becoming more prevalent with CAD/CAM systems trying to replicate clinician best practice. Hands-off casting is where a pressurised vessel records the shape and volume that the residual limb takes. This method relies on the principle of hydrostatic load bearing whereby load is distributed across the residual limb more uniformly.^9^ Theoretically, under this condition, pressure gradients and peaks are reduced, and less tissue shear generated.

Both hands-on and hands-off socket designs can achieve satisfactory clinical results.^7,8^ During prescription, interface fit is often assessed using tools that rely on user feedback and mobility performance testing.^3,10^ However, to develop more appropriate interface technology that is less likely to cause residual limb discomfort and injury, a better understanding of how socket design affects the mechanical conditions of the residual limb tissues is needed. Presently, researchers often measure interface pressure to compare socket designs.^11–13^ While indicative of the mechanical conditions at the skin surface, interface pressure cannot be used to understand the mechanical conditions of the internal tissues.

To go beneath surface level and get a better understanding of body device interface fit, tissue strain has been proposed as an important metric owing to its significance in soft tissue injury aetiology.^14–16^ Within the tissues of the residual limb, strain can cause cellular death through direct mechanical insult, ischemia, reperfusion, and impaired lymphatic drainage.^17^ Researchers have presented tools to interrogate the internal mechanical conditions of soft tissues, within prosthetics and more widely the rehabilitation field.

Finite Element Analysis (FEA) of the residual limb allows internal mechanical conditions to be studied.^18^ As the complexity of FEA models have increased, and more accurate material models and boundary conditions included, more confidence can be placed in these results. However, inherent limitations remain including the lack of material models that sufficiently accurately represent the biological materials of the residual limb. Furthermore, state of the art models demand significant computing resource and take a long time to set-up and complete making it currently unviable to conduct for large populations or in clinical settings.^19^

Digital Image Correlation (DIC) and Digital Volume Correlation (DVC) have also been proposed as tools to investigate residual limb tissue strains. 3D-DIC was used to record residual limb surface deformation which could then be used to calculate gross tissue deformation.^20^ However, this can only be conducted while no socket is worn, and the position of internal structures cannot be recorded. Rankin et al. manufactured an analogue residual limb consisting of bone structures and an elastomer filled with sand particles to represent the soft tissues.^21^ DVC of the analogue residual limb, using Computer Tomography (CT) imaging, was then performed while wearing two socket types. The authors suggested that the clinical translatability of this method was poor and questions remain about how the sand particles affect the behavior of the elastomer, and how representative this composite is of biological tissue.

Researchers have previously used the bone to skin surface distance when assessing body interfacing assistive technology designs in 2D^22^ and 3D.^23–25^ Changes in bone to skin distance infer the occurrence of tissue deformation. To the best of our knowledge, in the field of prosthetics, bone to skin distance in 3D has not been used as a tool to assess socket fit.

This report details a methodology to calculate, present, and evaluate the distance between the internal bony surface and skin surface of a residual limb. Using this information, interface designs can then be compared. As a pilot study, this methodology is used to compare a hands-on Patellar Tendon Bearing (PTB) socket and hands-off pressure cast socket for a single transtibial prosthesis user using secondary Magnetic Resonance Imaging (MRI) data.

## METHODOLOGY

### From MRI data to 3D STL Models

**Figure 1:**
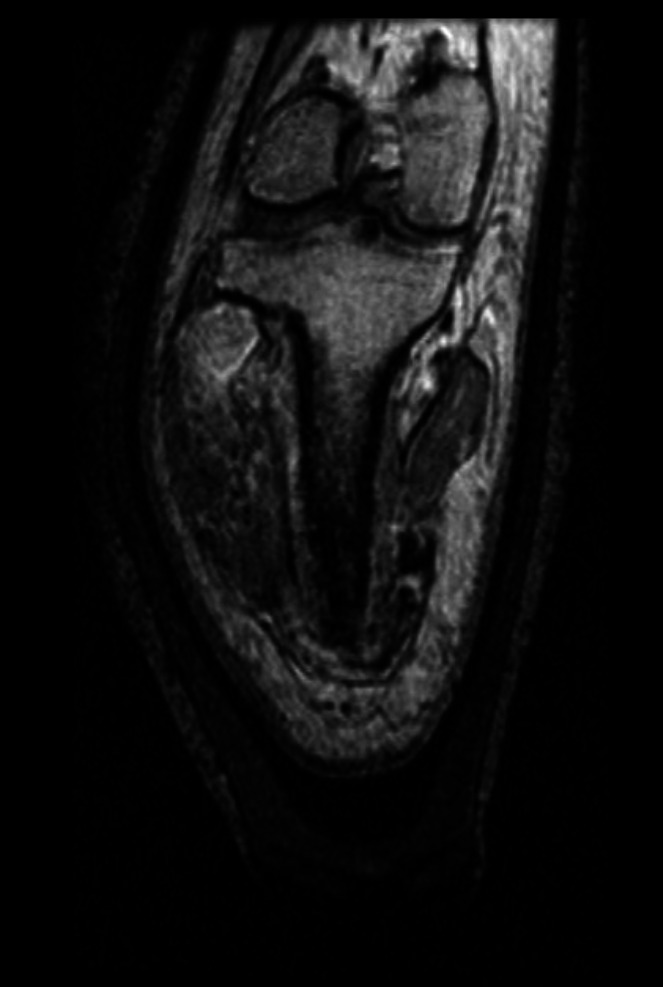
MRI Image in coronal plane of participants residual limb showing femur, tibia, and soft tissues.

MRI images of one person with unilateral transtibial amputation were retrieved from a secondary dataset to use in this study (**Figure 1**).^26^ The MRI data used was randomly selected from 12 potential participants. All data had been previously anonymised, and hence ethical approval was not required. Two MRI datasets were used from the same participant; one of the participant's residual limb while wearing a hands-on cast interface and one while wearing a hands-off cast interface. The MRI parameters used were as follows; field intensity 3 T, repetition time 6.9 s, time of echo 1.5 s, inversion time 500 ms, Bandwidth 31.25 KHz, flip angle 12 deg, matrix 256 × 256, slice thickness 1.2 mm, voxel dimensions 1.17 × 1.17 × 0.6 mm, and a 1-signal average.^26^ The residual limb was fixed in position during the scanning procedure by placing the patellar in a knee-cap receptacle and harnessing the thigh using pads and strapping.

The MRI data was imported into 3D slicer 5.2.2 medical image processing software.^27^ The following anatomical structures were segmented and extracted from the MRI datasets:

- **Soft Tissue**
- **Meniscus and Patellar Tendon**
- **Femur**
- **Patella**
- **Tibia**
- **Fibula**

Segmentation was performed semi-automatically using the ‘grow from seeds’ tool and then manually cleaned. The soft tissue segmentation included the skin, muscles, adipose, and connective tissues of the residual limb. The meniscus and patellar tendon were not included in the soft tissue structure as these are taken to be internal bodies that should be included in the hard tissue structure. The meniscus and patellar tendon were segmented together because of the challenge of processing individually. The other bony structures were segmented separately. Models were exported as stereolithography (STL) surface models.

**Figure 2:**
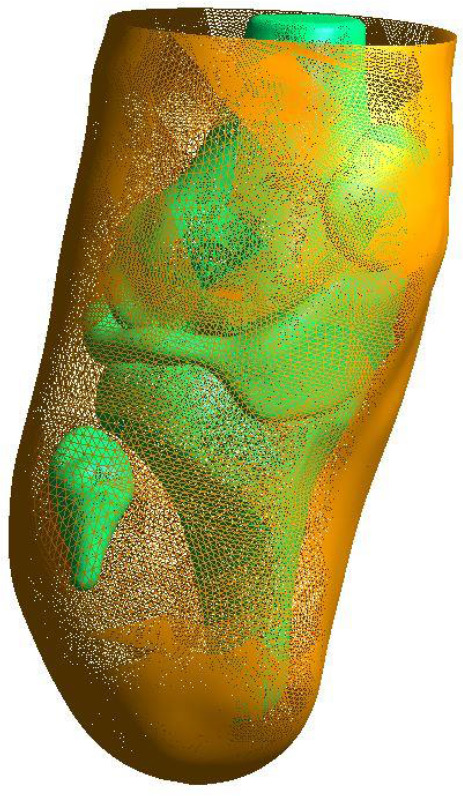
STL model of participants residual limb. Hard tissue surface is shown in green. Skin surface is shown in orange.

For each scan, the bone structures and meniscus/patellar tendon assembly were compiled into one STL file using Gmsh© model meshing software.^28^ This structure is hereby referred to as the hard tissue surface. This structure formed the surface for the nearest distance to the skin surface to be calculated from. The skin surface, being the outer surface of the soft tissue segmentation, was kept as a separate model (**Figure 2**).

The internal bone surface model was trimmed 50mm proximally to the most distal point on the femoral condyles. This represented a distance more proximal than the most proximal point on the socket brim across both interface types, ensuring all tissue encapsulated by an interface was included. The trim command was repeated for the skin surface using the same trim plane with reference to the global coordinate system.

CloudCompare software was used to calculate the distances between the STL vertices on paired surfaces using the nearest neighbour distance approach. For the first method of analysis, for each vertex on the internal bony surface, the corresponding nearest vertex on the skin surface is found and distance computed. For the second method of analysis, the hands-on and hands-off skin surfaces are registered and then distances between surfaces calculated. A description of each method is provided below:

### Internal bony surface to skin surface nearest distance; comparison between interface types.

For each socket type, the distance from every vertex on the internal bony surface to the nearest vertex on the skin surface is computed. A histogram of the distribution of surface-to-surface distances is retrieved from CloudCompare with the nearest distances split into 100 intervals. This process is repeated for both socket types. The number of nearest distance measurements for each socket type is then normalised to correct for discrepancies in the number of vertices of each STL file. The histogram for the hands-on and hands-off sockets are then combined into one graph.

### Hands-on interface vs. hands-off interface; skin surface visual comparison.

Using Gmsh^©^ software, a new STL file is created for both socket types that contains the skin surface and tibia surface only. These are loaded onto the CloudCompare interface. Next, the skin surface is separated from the tibia surface using the segment tool. Once complete, the tibia models are registered by aligning 10 manually selected equivalent point pairs. To ensure good registration, points are selected at key anatomical features that are easy to identify, and also at the extremities of the bone models. Finally, the tibia models are finely registered using CloudCompare's inbuilt fine registration tool. The quality of registration is quantified by calculating the absolute and average Hausdorff distances, a measure of similarity between shapes. The skin surfaces are then reinstated, and the nearest neighbour method is used to calculate the distance between both skin surfaces. The results are displayed as a heat map on the original skin surface. This process is then repeated with the other interface type as the reference surface and heat map inverted. The skin surfaces from both interface types are overlaid to allow the difference in surface topology to be visualised.

## RESULTS

### Internal bony surface to skin surface nearest distance; comparison between interface types (Figure 3).

**Figure 3:**
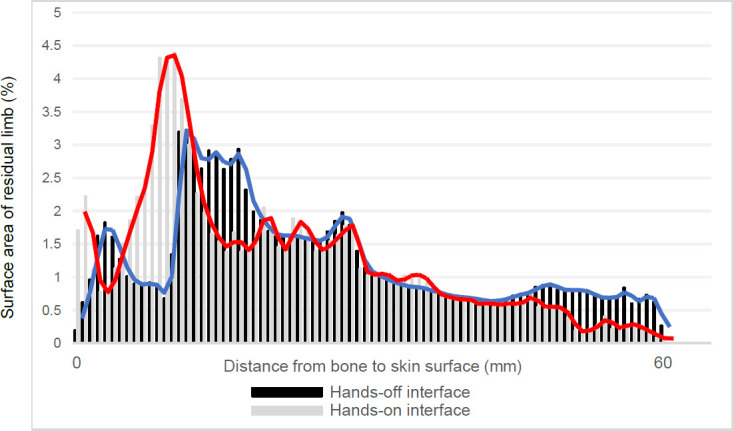
This graph shows percentages of the skin surface that are within a certain distance from the bone surface. The hands-off socket is shown in black/blue, and the hands-on socket is shown in gray/red.

The nearest distance between the internal bony surface and skin surface, for every surface vertex, was calculated for both interface types: while wearing a hands-on interface and while wearing a hands-off interface. For the hands-on and hands-off interfaces, the maximum nearest distances between the internal hard tissue structure and skin surface were similar at around 60mm. The smallest nearest distances between the internal hard tissue structure and skin surfaces were in the order of 1mm for both sockets. For the hands-on socket, 4.4% of the skin surface was between 7.8mm and 8.4mm from the internal bony surface, representing the most common distance for this condition. For the hands-off socket, 3.2% of the skin surface was between 9mm and 8.6mm from the internal hard tissue structure, representing the most common distance for this condition. When comparing the hands-on interface to the hands-off interface, the hands-on interface has a higher peak that is positioned further left along the x-axis. In contrast, the hands-off interface has a lower peak that is positioned further right along the x-axis. A greater proportion of the hands-off interface skin surface is found to be further away from the internal bony surface as seen at the right extremity of the x-axis (**Figure 3**).

### Hands-on interface vs. hands-off interface; skin surface visual comparison (Figure 4).

After registration of the tibia models, the absolute bidirectional Hausdorff distance was 2.81mm and the average bidirectional Hausdorff distance was 0.43mm.

The resulting skin surface geometries for the hands-on and hands-off interface types were compared. Areas highlighted in red show where the hands-on interface has a more compressive fit than the hands-off interface. Areas highlighted in blue show where the hands-off interface has a more compressive fit than the hands-on interface. The hands-on interface has a more compressive fit at the distal end of the residual limb, across the patellar tendon, above the medial and lateral femoral epicondyles. The hands-off interface has a more compressive fit more proximally on the residual limb, specifically on the anterior aspect.

## DISCUSSION

The methods proposed in this report indicate deformation of residual limb soft tissues. The first method of analysis can be used to help understand how uniformly an interface design deforms residual limb soft tissue. The second method of analysis can be used to visualise where on the residual limb a socket design is more compressive and where the fit is more relaxed. This information can be used as a further tool to improve interface fit by providing a better understanding of how interface designs affect the soft tissues of the residual limb.

### Internal bony surface to skin surface nearest distance; comparison between interface types.

The histogram profile is suggestive of interface fit. While wearing the hands-on interface, the higher peak of the histogram, positioned further left along the x-axis, points to higher levels of deformation, and hence strain, being induced in a larger volume of the residual limb than compared to the hands-off interface. In contrast, for the hands-off interface, the lower peak positioned further right along the x-axis suggests that this interface delivers a more uniformly compressive fit.

A shorter distance between the internal bony surface and skin surface describes a smaller depth of soft tissue. At these smaller distances, less absolute deformation is required to induce higher, possibly dangerous, levels of tissue strain. There is a greater volume of residual limb in the first three intervals of the histogram (<1.8mm soft tissue depth) for the hands-on condition than for the hands-off condition. While a comparison to the no-socket condition cannot be made for this study, the more compressive fit over areas of shallow tissue depth for the hands-on condition indicates that high tissue strain levels will be generated.

### Hands-on interface vs. hands-off interface; skin surface visual comparison.

**Figure 4:**
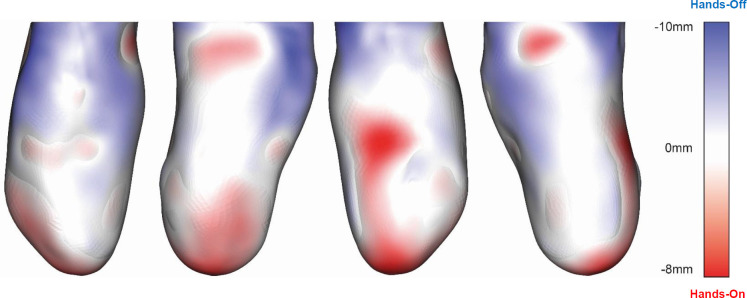
Comparison of skin surface while wearing hands-on and hands-off interface using method 2. Areas where hands-on interface provides a more compressive fit when compared to the hands-off interface are shown in red. Areas where hands-off interface provides a more compressive fit when compared to the hands-on interface are shown in blue.

Much of what is shown in **Figure 4** agrees with hands-on and hands-off interface fit that has been reported in the literature. The classic features of the hands-on PTB socket, such as the patellar tendon bar, popliteal depression, and medial and lateral supracondylar depressions can be easily identified.^29^ The longer residual limb length while wearing the hands-off cast is also in agreement with previously reported literature.^26^ This suggests that the participants residual limb, and hands-on and hands-off casts, were fairly typical. Surprisingly there is no observable difference in socket geometry over the fibular head, a sensitive feature of the residual limb where offloading is commonly included as part of hands-on design.^30^ However, due to the small tissue depth over the fibular head, whether this methodology is sensitive enough to pick up any difference should be questioned. Interface pressure is commonly visualised using heatmaps^31,32^ but deformation or topology, as reported here, is less common with few examples.^29^

### Considerations and Future Work

The nearest distance between the internal bony surface and skin surface, and skin surface to skin surface comparison between interface types, indicates deformation of residual limb soft tissues. However, the distribution of strain within residual limb tissues cannot be ascertained. Furthermore, whether a structure is at higher risk of tissue damage is not taken into consideration. Different tissues have different mechanical properties, discomfort or damage thresholds, and the makeup of different tissue types in an area of residual limb is not considered.^18^ There are also amputation specific factors that aren't included such as the presence of scarring^33^ or an already compromised vascular system.

The process of converting the MRI scans into STL models requires multiple steps, during which, some geometric accuracy is likely lost. This is especially pertinent when investigating structures with smaller soft tissue depths, nearing the resolution of the MRI data, where a loss in accuracy will have a big impact on the resulting error. High definition MRI data allows for more accurate segmentation. For future studies, what counts as sufficiently high-resolution MRI data, must be defined. This should be noted as a significant limitation in this pilot study as the quality of the secondary MRI data was poor.

During ambulation, prostheses are loaded dynamically and movement of the residual limb bones with respect to the socket wall occurs.^34^ The methodology presented in this report only looks at a static loading condition. Using this methodology to compare different levels of static loading while wearing the same interface would go some way to understanding how different interface designs influence residual limb soft tissue deformation during prostheses use.

Comparing the geometry of the residual limb while wearing prosthetic devices against wearing no device would be of interest. Unfortunately, MRI images of the participant's residual limb with no interface donned were not available for this study. Collecting MRI data of the residual limb without significant distortion of the tissues due to gravity is challenging. For this reason, imaging the residual limb while wearing only a liner to reduce sagging might be better suited as a baseline condition for comparison.

This pilot study only reports on a single participant and therefore the results cannot be generalised to a wider population. Future work should focus on validating this methodology by comparing results to a range of other measures, for a larger study population to look for patterns in tissue deformation caused by interface fit, and including the liner-only condition.

## CONCLUSION

The methodology presented in this report may give researchers a further tool to better understand how interface designs effect the soft tissues of the residual limb. When used to compare the residual limb of a single participant while wearing a hands-on and hands-off interface, differences in the distribution of nearest internal bony surface to skin surface distances were observed. A further study comparing interface designs to no prosthesis or liner only condition should be completed, upon which it will be possible to have more confidence in this technique. Attaining a complete understanding of the biomechanics of the residual limb and its interaction with prosthetic devices remains an unsolved challenge that limits the advancement of prosthetic interface design.

## DECLARATION OF CONFLICTING INTERESTS

The authors report that there are no conflicts of interest to declare.

## AUTHORS CONTRIBUTION

**Thomas Arnstein** conceived the idea, conducted the research, and drafted the manuscript, while **Arjan Buis** provided supervision. Both authors actively participated in the discussion of results and contributed to the manuscript's revisions.

## SOURCES OF SUPPORT

EPSRC GRANT NUMBER: EP/S02249X/1
